# Exercise training reduces cardiac fibrosis, promoting improvement in arrhythmias and cardiac dysfunction in an experimental model of chronic chagasic cardiomyopathy

**DOI:** 10.3389/fphys.2025.1558678

**Published:** 2025-04-28

**Authors:** Alex Cleber Improta-Caria, Carolina Kymie Vasques Nonaka, Pâmela Santana Daltro, Carine Machado Azevedo, Breno Cardim Barreto, Gisele Batista Carvalho, Juliana Fraga Vasconcelos, Bruno Solano de Freitas Souza, Simone Garcia Macambira, Milena Botelho Pereira Soares

**Affiliations:** ^1^ Multicentric Program of Post-Graduate in Biochemistry and Molecular Biology, Federal University of Bahia (UFBA), Salvador, Bahia, Brazil; ^2^ Center for Biotechnology and Cell Therapy, São Rafael Hospital, Salvador, Bahia, Brazil; ^3^ Laboratory of Biochemistry and Molecular Biology of the Exercise, University of Sao Paulo (USP), Sao Paulo, Brazil; ^4^ D’Or Institute for Research and Education (IDOR), Salvador, Brazil; ^5^ Gonçalo Moniz Institute, Oswaldo Cruz Foundation (FIOCRUZ), Salvador, Bahia, Brazil; ^6^ Department of Biochemistry and Biophysics, Health Sciences Institute, Federal University of Bahia, Salvador, Bahia, Brazil; ^7^ Technology Institute of Health, SENAI CIMATEC, Salvador, Bahia, Brazil

**Keywords:** chronic chagasic cardiomyopathy, exercise training, immunomodulation, arrhythmias, cardiac fibrosis

## Abstract

**Background:**

Chagas disease, caused by the parasite *Trypanosoma cruzi*, is associated with inflammation and fibrosis, which characterizes chronic Chagasic cardiomyopathy (CCC). CCC manifests as arrhythmias, hypertrophy or dilation of the left ventricle, and it may progress to heart failure. Therefore, interventions are needed to slow the progression of CCC. Aims: We investigated the effects of exercise training in an animal model of CCC.

**Methods:**

C57BL/6 mice infected with *Trypanosoma cruzi* were submitted to a progressively treadmill exercise training protocol. The cardiac function was evaluated by echocardiogram and electrocardiogram. RT-qPCR and morphometric analyses were performed on samples of cardiac tissue to quantify inflammation and fibrosis.

**Results:**

EKG analysis confirmed that all infected mice developed arrhythmias, with different degrees of severity. Exercise improved arrhythmias in 43.75% of chagasic trained mice, and the remaining mice did not show any alteration in EKG. The untrained chagasic group had no improvement in arrhythmias. The ventricular compliance in chagasic trained mice increased, as revealed by the reduction in isovolumetric relaxation time when compared to untrained mice. Exercise induced the reduction of gene expression of TGF-β, TNF-α, IL-1β, IL-6 and MMP-9 and reduced fibrosis in the heart tissue of chagasic mice.

**Conclusion:**

Exercise reduced fibrosis in the heart and skeletal muscle, favoring the improvement of arrhythmias, and augment of cardiac complacency in mice with CCC, in addition to decreasing the expression of profibrotic and proinflammatory genes in the heart.

## 1 Introduction

Chagas disease, caused by the protozoan *Trypanosoma cruzi* is considered one of the twenty neglected tropical diseases and represents a major health problem in Latin America, where it is estimated 6 to 7 million infected individuals (Navarro et al., 2022). The symptomatic chronic forms occur many years or decades after the acute phase. The most severe symptomatic form is chronic chagasic cardiomyopathy (CCC), a cardiac disturbance developed in 30% of infected people that progresses to myocardium contractility dysfunction, severe arrhythmias, and sudden death (Cunha-Neto and Chevillard, 2014). These abnormalities are due to intense myocarditis and fibrosis (Meira et al., 2019) that can culminate in heart failure (HF) (Bocchi et al., 2017).

Recently, the CCC was subdivided into five forms, according to signs, symptoms, and cardiac evaluations performed by electrocardiography, echocardiography, and chest X-ray (Bocchi et al., 2017). The only definitive therapeutic option for patients with severe HF is heart transplantation, which is a complex procedure with high cost, donor organ shortage, and complications due to the use of immunosuppressive drugs after transplantation, which may reactivate the latent infection (Elalouf, 2023). Therefore, the majority of CCC patients are only eligible for symptomatic treatment. Therefore, a strategy that interferes with the progression of CCC and/or improves cardiac function in patients with advanced heart disease is needed.

Exercise training (ExT) is suggested, since the 20th century, as a tool to prevent cardiac diseases (Haskell and Fox, 1968). Regular ExT prevents deterioration and improves cardiac performance in patients with heart diseases, mainly from ischemic etiology and chronic HF (Sachdev et al., 2023). ExT acts as a non-pharmacological therapeutic agent to prevent disease and promote cardiovascular health through molecular signaling pathways (Caria et al., 2018; Improta-Caria and Aras, 2021; Improta-Caria, 2022) involved in organism adaptations to ExT (Fernandes et al., 2011; Fernandes et al., 2012; Improta-Caria et al., 2024). Other studies suggested that the effects of ExT on the cardiac system are mediated through autonomic response (Fiuza-Luces et al., 2018). ExT can also contribute to health, quality of life, lifespan, and a more favorable disease progression through different mechanisms (De Sousa and Improta-Caria, 2022), including immunomodulatory processes (Fernandez et al., 2018).

Although the benefits of ExT in chronic HF and other diseases are well established (Piña et al., 2003; Cattadori et al., 2018), little is known for CCC. Few clinical trials investigated the effects of ExT on the progression of CCC and on health and quality of life. Using different protocols of training and measurement of parameters, all studies demonstrated that regular ExT was feasible, safe, and tolerable by patients with CCC, promoting improvement in cardiac functional capacity and quality of life (Lima et al., 2010; Fialho et al., 2012; de Souza and Improta-caria, 2022). Furthermore, the Brazilian Society of Cardiology guidelines state that the practice of ExT is important to improve several clinical parameters of patients with CCC (Marin-Neto et al., 2023).

This highlights the potential benefit of implementing a standardized ExT protocol as part of the therapeutic management of patients with CCC. However, the effects of ExT on pathophysiological processes in the heart such as fibrosis and inflammation, as well as on arrhythmias and cardiac dysfunction in individuals with CCC have been poorly elucidated. Therefore, the present study aimed to evaluate the adaptations induced by ExT on cardiac fibrosis and inflammation, as well as on arrhythmias and cardiac dysfunction due to *T. cruzi* infection, using an experimental model of CCC in mice.

## 2 Materials and methods

### 2.1 Animals

Two-month-old female C57BL/6 mice (n = 39) used in this study were kept in the animal facilities at the Center for Biotechnology and Cell Therapy (São Rafael Hospital, Salvador, Bahia, Brazil). The size of the experimental group was based on our previous studies (Garcia et al., 2005; Macambira et al., 2009; Vasconcelos et al., 2013) in which the number of animals per group varied between 6 and 10 mice depending on the number of analyses to be conducted. The size of the experimental group in all studies was calculated and confirmed using the WinPepi software. The animals were divided into three experimental groups: uninfected non-trained mice (n = 7 – CTL), infected non-trained mice (n = 16 – Sed CCC), and infected trained mice (n = 16 – ExT CCC). The mice were given a rodent diet and water *ad libitum* in a 12 h-light-dark cycle (6:00 a.m. - 6:00 p.m.) under ideal temperature and humidity conditions and continuous airflow. At the end of the study, all animals were euthanized under anesthesia by intraperitoneal injection of xylazine (Sedomin; König do Brasil, São Paulo, Brazil) at 10 mg/kg body weight and ketamine (Vetanarcol; Konig, Santana de Parnaíba, Brazil) at 100 mg/kg body weight and handled according to the National Institutes of Health guidelines for ethical use of laboratory animals. The experimental protocol was approved by the Ethics Committee on Animal Use of São Rafael Hospital (Protocol Number 001/2015).

### 2.2 Trypanosoma cruzi infection

Thirty-two mice were infected via intraperitoneal injection with 1,000 trypomastigote forms of the Colombian strain of *T. cruzi*, obtained through *in vitro* infection of the LCC-MK2 cell line. Parasitemia was assessed by counting the number of trypomastigotes in peripheral blood aliquots up to 60 days post-infection to confirm the infection.

### 2.3 Electrocardiogram (EKG) analysis

EKG acquisition was performed in bipolar I and II leads using the Bio Amp PowerLab System (PowerLab 2/20; ADinstruments, Castle Hill, NSW, Australia). All mice were anesthetized with inhaled isoflurane (0.5%) to obtain the records. The data was acquired for computer analysis using Chart 5 for Windows (PowerLab). Records were bandpass filtered (1–100 Hz) to minimize environmental signal disturbances. The sampling rate was 1 kHz. The EKG analysis included heart rate, PR interval, P wave duration, QT interval, QTc, and arrhythmias. Wave durations (ms) and heart rate were calculated automatically by the software after the cursors were placed. The QTc was calculated as the ratio of QT interval by square roots of RR interval. The animals were evaluated 8 months post infection and 1 week after the end of ExT protocol.

### 2.4 Echocardiography (ECHO) analysis

Animals were anesthetized with inhaled isoflurane (0.5%). Transthoracic ECHO was performed on supine-positioned mice maintained on a thermo-regulated plate (37°C) to acquire images in different acoustic windows by using the Vevo 770 Ecosystem (Visual Sonics, Toronto, Canada) equipped with a 30 MHz transducer (Model 707B RMV, Visual Sonics). ECHO analysis was performed using M-mode and B-mode image acquisition tools allowing for the visualization and measurement of left ventricular wall motion, anatomical structures, and hemodynamics parameters, thereby enabling the detection of morphological and functional alterations. Ventricular wall thickness and the inner diameter of the left ventricle during systole and diastole were determined from M-mode and B-mode images by measuring blood flow, using pulse Doppler hemodynamics. The function parameters evaluated were fractional shortening, ejection fraction, systolic volume, end-diastolic volume, and cardiac output. The animals were evaluated 8 months post-infection and 1 week after the end of the ExT protocol.

### 2.5 Aerobic ExT protocol

**FIGURE 1:**
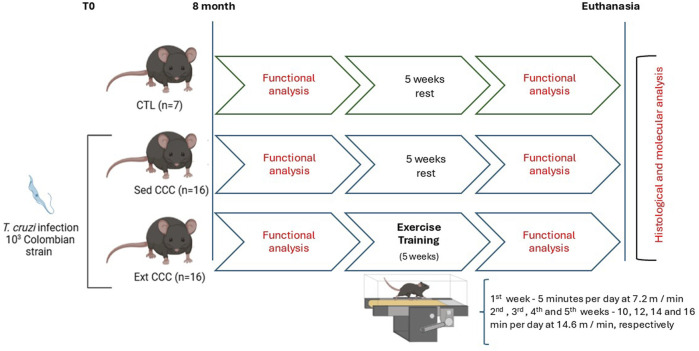
Exercise training protocol.

A motor-driven treadmill chamber for one animal (LE 8700; Panlab, Barcelona, Spain) was used to exercise the mice. The speed of the treadmill and the intensity of the shock (mA) were controlled by a potentiometer (LE 8700-treadmill control; Panlab). Room air was pumped into the chamber at a controlled flow rate (700 mL/min) by a chamber airsupplier (Oxylet LE 400; Panlab). The mice (n = 39) were divided in three experimental groups: CTL, Sed CCC, and ExT CCC. The protocol used was adapted from Souza et al., 2015. The ExT program lasted a total of 5 weeks, with progressive intensity, 5 times a week, with the 1st week being adaptive: 5 min per day at 7.2 m/min. In the 2^nd^, 3^rd^, 4^th^, and 5^th^ weeks, there were 10, 12, 14 and 16 min per day at 14.6 m/min, respectively (Figure 1).

### 2.6 Morphometric analyses

All mice were euthanized upon completion of the ExT program as described before. Half of the heart and gastrocnemius muscle were collected from each mouse and then fixed in 10% buffered formalin for histological processing. A longitudinal section was made in the heart subjected to morphometric analysis, allowing the evaluation of the atrial and ventricular regions. Tissue sections of 4 µm were made to create histological slides after paraffin embedding. The slides were submitted to standard hematoxylin and eosin (H&E) staining to quantify inflammatory cells and by Sirius Red with Fast Green to evaluate the percentage of fibrosis. Heart and skeletal muscle slides were analyzed by light microscopy. Ten areas (400x) containing the highest number of inflammatory cells were photographed from every mouse of each group, as described before (Vasconcelos et al., 2013). The quantification of the percentage of fibrosis was carried out on slides stained with Sirius red (200x) where ten areas were also photographed looking for regions that showed greater collagen deposition, excluding regions of blood vessels. Images were digitized using a color digital video camera (CoolSnap, Photometrics, Montreal, QC, Canada) adapted to a BX41 microscope (Olympus, Tokyo, Japan). Morphometric analyses were performed using the software Image-Pro Plus v.7.0 (Media Cybernetics¸ San Diego, CA, United States). All the analyses were performed in a blinded fashion.

### 2.7 Quantitative reverse transcription polymerase chain reaction (RT-qPCR) analysis

After the collection, half of the hearts were used for RNA extraction. RNA was extracted from heart tissue with TRIzol (Invitrogen), and the concentration was determined by photometry. A High-Capacity cDNA Reverse Transcription Kit (Applied Biosystems) was used to synthesize complementary DNA (cDNA) from 1 µg of RNA, according to the manufacturer’s recommendations. Production of cDNA for RNA expression analysis and amplification were performed by Real-Time polymerase chain reaction (PCR) using TaqMan Gene Expression Assay for TNFα (*Mm00443258_m1*), TGFβ1 (*Mm00441724_m1*), IL-1β (*Mm00434228_m1*), IL-6 (*Mm00446190_m1*); MMP-9 (*Mm00442991_m1*), and TIMP-1 (*Mm00441818_m1*). All reactions were run in duplicate, using an ABI 7500 Real Time PCR System (Applied Biosystems), under standard thermal cycling conditions. A non-template control (NTC) and non-reverse transcription controls (No-RT) were also included. The samples were normalized with GAPDH gene (endogenous control). The threshold cycle (2^−ΔΔCT^) method of comparative PCR was used to analyze the results (Schmittgen and Livak, 2008). Data was analyzed using GraphPad software version 6.

### 2.8 Statistical analysis

Data were expressed as mean ± standard error of the mean (SEM) for the number of animals in each group. Prior to statistical analyses, data normality was assessed using the Shapiro-Wilk test, confirming a normal distribution and homogeneity of variances. Student’s t-test was used for comparisons between two groups, while analysis of variance (ANOVA) was applied for comparisons among three or more groups, followed by Bonferroni’s *post hoc* test. Differences were considered statistically significant for P < 0.05. Statistical analyses were performed using GraphPad Prism 8.0 software (San Diego, CA, United States).

## 3 Results

### 3.1 Aerobic ExT attenuates the severity of cardiac arrhythmias

Eight months after *T. cruzi* infection, all Sed CCC and ExT CCC mice had cardiac arrhythmias with different degrees of severity, such as first-degree atrioventricular block, intraventricular conduction disturbance, monomorphic ventricular tachycardia, polymorphic ventricular tachycardia, and total atrioventricular block, whereas no disturbances were found in CTL mice (Table 1).

**TABLE 1:** Arrhythmias in chagasic mice before and after ExT.

Experimental Groups	Arrhythmia											
	NSR		1^ST^ AVB		IVCD		MVT		PVT		T AVB	
	PRE	POST	PRE	POST	PRE	POST	PRE	POST	PRE	POST	PRE	POST
Sed CTL (7)	7	7	0	0	0	0	0	0	0	0	0	0
ExT CCC (16)	0	0	2*	8	2*	2	8	3	4	1	2	4
Sed CCC (16)	0	0	2**	2	2**	2	2	3	7	7	4	3

Presence of arrhythmias in Chagasic mice before (PRE) and after (POST) ExT. The table presents the number of mice exhibiting different types of arrhythmias in each experimental group: CTL (Non-trained control mice), ExT CCC (Trained Chagasic mice), and Sed CCC (Non-trained Chagasic mice). Normal Sinus Rhythm (NSR). Arrhythmias analyzed include: First-Degree Atrioventricular Block (1st AVB), Intraventricular Conduction Disturbance (IVCD), Monomorphic Ventricular Tachycardia (MVT), Polymorphic Ventricular Tachycardia (PVT), and Total Atrioventricular Block (T AVB). The numbers indicate the count of animals presenting each type of arrhythmia before and after the ExT protocol. *The same trained Chagasic mice. **The same non-trained Chagasic mice.

**FIGURE 2:**
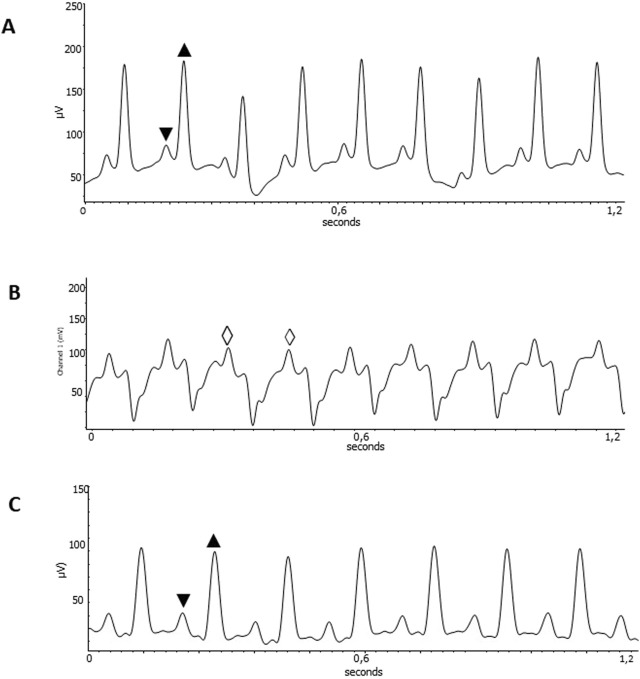
Improvement of cardiac disturbances after ExT. EKG recordings from healthy C57Bl6 mice uninfected, Heart rate of 511 bpm; PR interval 36 ms; QT interval of 50 ms **(A)**; *ExT CCC* mice pre-training, Heart rate of 418 bpm **(B)**; and *ExT CCC* mice post-training, Heart rate of 455bpm; PR interval 56 ms; QT interval of 60 ms **(C)**. ▼ P wave; ▲QRS complex; ◊ sustained Monomorphic ventricular tachycardia. Wave amplitude in µV. Time in seconds.

**FIGURE 3:**
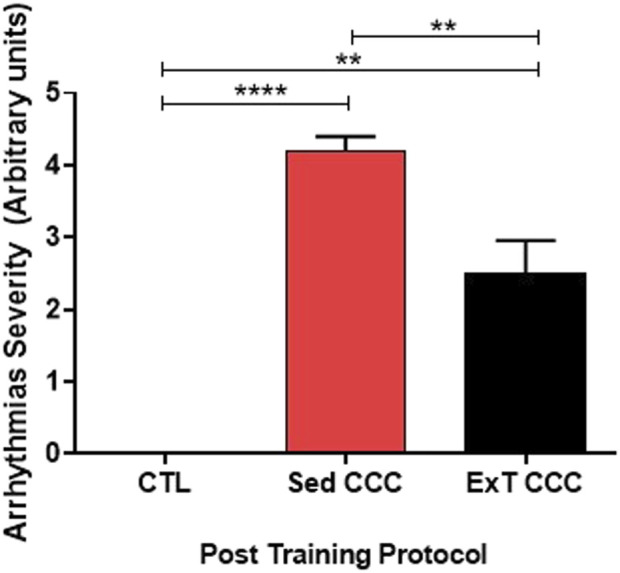
Arrhythmias severity. ExT was able to reduce the severity of arrhythmias.

ExT CCC mice had an improvement in the severity of arrhythmias. Eight animals from this group developed monomorphic sustained ventricular tachycardia due to the infection, as determined before ExT. Four mice reverted to first-degree atrioventricular block after ExT, three mice had no alteration, and one mouse evolved to total atrioventricular block (Table 1; Figure 2). Other four CCC animals had polymorphic sustained ventricular tachycardia before ExT, and two reverted to first-degree atrioventricular block, one mouse evolved to total atrioventricular, and another one remained stable in polymorphic sustained ventricular tachycardia after ExT, demonstrating that ExT was able to attenuate the severity of arrhythmias (Figure 3).

Sed CCC mice had the same arrhythmias recorded in ExT CCC. However, no improvement was observed over time in Sed CCC mice, except for one mouse that developed total atrioventricular block in the period corresponding to pre-training that had monomorphic sustained ventricular tachycardia with atrioventricular dissociation.

### 3.2 Cardiac remodeling due to chronic chagasic cardiomyopathy

**FIGURE 4:**
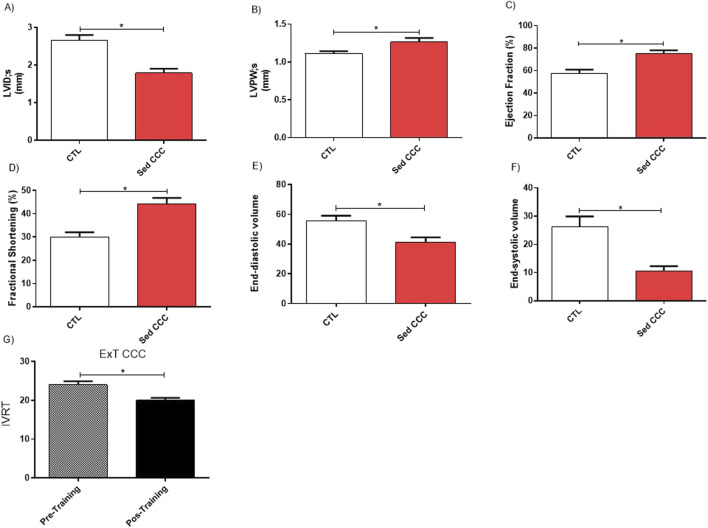
Echocardiography functional assessment. Echocardiographic analyses were performed before and 1 week after the end of ExT in control uninfected mice and infected mice. The comparative analyses between control and chagasic mice before training revealed significantly differences in Ventricular Internal Diameter during systole **(A)**; Posterior Wall Thickness during systole **(B)**, Ejection Fraction **(C)**; Fractional Shortening **(D)**; End-diastolic Volume **(E)** and End-systolic volume **(F)** and Isovolumetric Relaxation Time **(G)**. Values are expressed as mean ± SEM. *p ≤ 0.05.

Before the ExT protocol, the ECHO parameters obtained from CTL and Sed CCC mice were compared (Figures 4A–F). The measurement of internal ventricular diameter and posterior wall thickness demonstrated that chronic Sed CCC mice developed cardiac concentric hypertrophy. The pseudo normalization phenomenon was suggested by higher ejection fraction and fractional shortening and lower end-diastolic and end-systolic volumes in Sed CCC. After the end of ExT protocol, the isovolumetric relaxation time was shortened in ExT CCC compared to Sed CCC mice which indicates an increase in ventricular complacency and improvement in hemodynamic function (Figure 4G).

### 3.3 Aerobic ExT reduces heart and skeletal muscle fibrosis in chagasic mice

**FIGURE 5:**
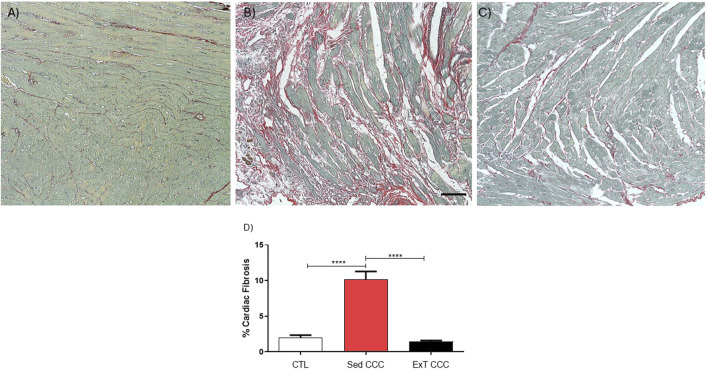
Histopathological analysis in heart sections. Heart sections stained with Picrosirius Red of CTL mice **(A)**, Sed CCC mice **(B)** and ExT CCC mice **(C)**. Morphometric analysis of the percentage of fibrosis area in heart sections **(D)**. Values are expressed as mean ± SEM. ****P < 0.0001.

**FIGURE 6:**
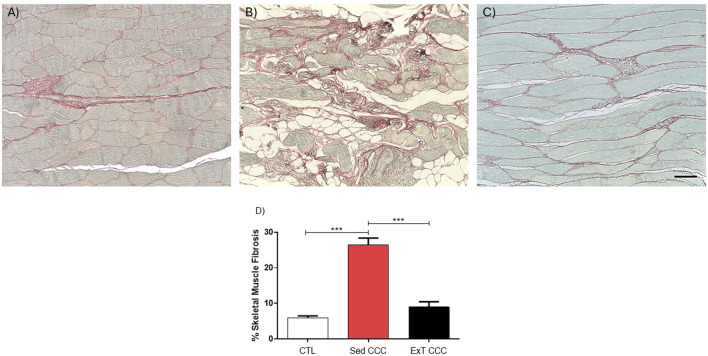
Histopathological analysis in skeletal muscle sections. Skeletal muscle sections stained with Picrosirius Red of CTL mice **(A)**, Sed CCC mice **(B)** and ExT CCC mice **(C)**. Morphometric analysis of the percentage of fibrosis area in skeletal muscle sections **(D)**. Values are expressed as mean ± SEM. ****P < 0.0001.

Next, we evaluated the effects of ExT on fibrosis and inflammation in the heart and in the skeletal muscle. Heart sections of ExT CCC group had a significant reduction in the percentage of fibrosis, as evaluated 1 week after the end of ExT, compared to Sed CCC (Figures 5A–D). In addition to the reduction in cardiac fibrosis, we also observed a fibrosis reduction in skeletal muscle from ExT CCC mice compared to Sed CCC (Figures 6A–D).

### 3.4 Aerobic ExT did not reduced the number of inflammatory cells in the heart and skeletal muscle in chagasic mice

**FIGURE 7:**
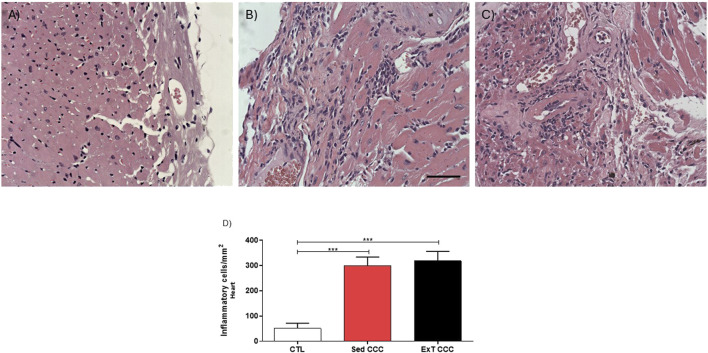
Quantification analysis of inflammatory cells in the cardiac tissue. Heart sections stained with H&E of CTL **(A)**, Sed CCC **(B)** and ExT CCC mice **(C)**. Inflammatory cells were quantified in heart sections and integrated by area **(D)**. Values are expressed as mean ± SEM. ***P < 0.001.

**FIGURE 8:**
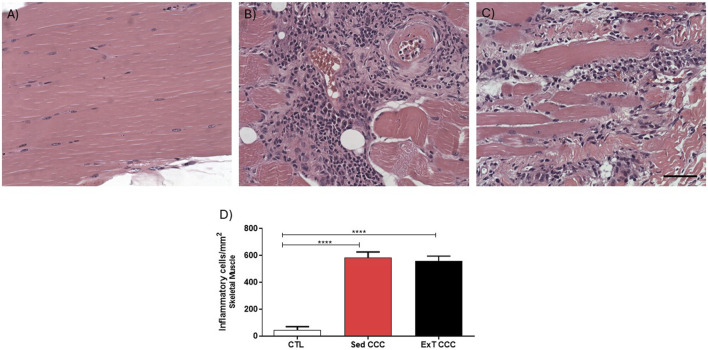
Quantification analysis of inflammatory cells in the skeletal muscle tissue. Skeletal muscle sections stained with H&E of CTL **(A)**, Sed CCC **(B)** and ExT CCC mice **(C)**. Inflammatory cells were quantified in skeletal muscle sections and integrated by area **(D)**. Morphometrical analysis Values are expressed as mean ± SEM. ****P < 0.0001.

The analysis of inflammatory cells showed that CCC groups had significantly higher numbers of inflammatory cells in the heart when compared to CTL mice (Figures 7A–D). However, the number of inflammatory cells was similar in Sed CCC versus ExT CCC mice (Figure 7D). In the same way as observed in the heart, the number of inflammatory cells in the skeletal muscle of the CCC groups was significantly higher than in the CTL group; and there was no difference between the Sed CCC and ExT CCC groups (Figures 8A–D).

### 3.5 Aerobic ExT reduced the expression of proinflammatory and profibrotic genes in the heart

**FIGURE 9:**
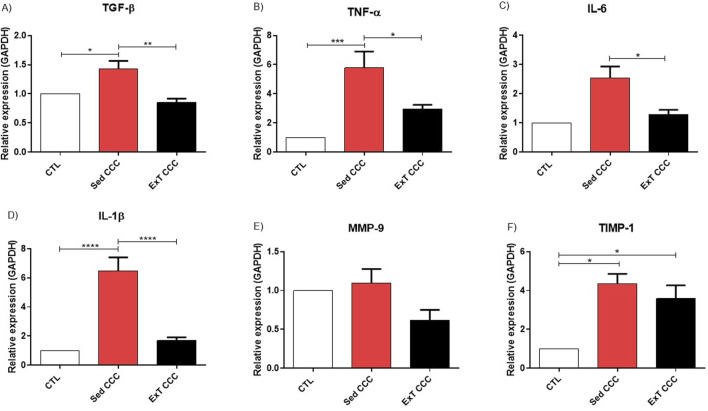
RT-qPCR in heart tissue. Representative values of TGF-β **(A)**, TNF-α **(B)**, IL-6 **(C)**, IL-β **(D)**, MMP-9 **(E)** and TIMP-1 **(F)**. Expression obtained 1 week after the end of ExT. Values are expressed as mean ± SEM of 6 mice/non-infected group; 8 mice/infected group). *p ≤ 0.05; **p ≤ 0.01; ***p ≤ 0.001.

Finally, the expression of genes related to inflammation and fibrosis was evaluated by quantitative reverse transcription PCR (RT-qPCR) in heart samples, after ExT. The levels of proinflammatory and fibrotic genes, such as transforming growth factor beta (TGF-β), tumor necrosis factor alpha (TNF-α), interleukin 1 beta (IL-1β), and tissue inhibitor of metalloproteinases-1 (TIMP-1) are increased in Sed CCC mice compared to CTL mice. ExT promoted a reduction in the expression of these genes to similar levels to CTL, in addition to reducing the expression of interleukin 6 (IL-6) gene compared to the Sed CCC group however, we did not observe a decrease in TIMP-1 expression when comparing ExT CCC with Sed CCC (Figures 9A–F).

## 4 Discussion

The improvement in cardiac function mediated by ExT has already been studied in cardiomyopathy of different etiologies, however, little is known about the effects of ExT on cardiac function in CCC. Few studies with patients with Chagas heart disease have shown that ExT or a cardiac rehabilitation program induces an increase in VO^2^ peak, improves the anaerobic threshold and maximum minute ventilation, promoting an improvement in the functional capacity of these patients (de Souza Nogueira Sardinha Mendes et al., 2020). In addition, ExT improves the mental health and quality of life of these people (Vieira et al., 2024), and can also be considered a good cost-effective strategy (Simões et al., 2022).

However, our study investigated the effects of ExT on functional parameters and at histological and molecular levels. We used a chronic chagasic model in C57BL/6 mice infected with a Colombian strain of *T. cruzi*. The murine model of long-term infection with *T. cruzi* to elucidate the pathogenesis of CCC has been established in our laboratory for a long time, leading to progressive cardiomyocyte loss, inflammation, fibrosis, electrophysiological abnormalities, arrhythmias, impairment of ventricular function and loss of physical exercise capacity (Soares et al., 2004; Garcia et al., 2005; Macambira et al., 2009; Vasconcelos et al., 2013). According to Kunstyr and Leuenberger (1975), the lifespan of C57BL/6 mice, estimated to range from 2 to 3 years under laboratory conditions, provides a suitable model for studying the long-term progression of infection in humans. This longevity enables the investigation of disease mechanisms and the evaluation of therapeutic interventions over an extended period, mimicking chronic disease progression.

Electrocardiographic analysis of infected C57BL/6 mice demonstrates functional cardiac abnormalities that correlate with the severity of chronic experimental Chagas cardiomyopathy (Garcia et al., 2005); Pereiraet al., 2014). The Colombian strain belongs to the TcI discrete typing unit (DTU) of *T. cruzi*, which is the most widespread and genetically diverse lineage of the parasite in the Americas (Hoyos Sanchez et al., 2024). Furthermore, this strain is well-suited for this study, as it has been shown to exhibit a strong tropism for myocardial and skeletal muscle tissues, resulting in severe injuries as the disease progresses (Camandaroba et al., 2006).

This study demonstrates that ExT exerts beneficial effects on cardiac function and structure in an experimental model of CCC. These improvements are primarily associated with a reduction in cardiac fibrosis in the left-sided cardiac tissue, as observed in the experimental group subjected to the aerobic ExT protocol. In the experimental model of CCC developed in mice, the left side of the heart is typically more affected than the right. Studies have shown that pathological changes, including fibrotic and inflammatory alterations, are generally more pronounced in the left ventricle and the left atrium of infected mice. This aligns with observations in human cases of Chagas disease, where left ventricular dysfunction is a common feature. Chronic infection with *T. cruzi* leads to a range of cardiac abnormalities, and the left side of the heart exhibits significant structural and functional impairments, such as cardiomegaly and left ventricular dilation, which are characteristic of the disease’s progression (Pereira et al., 2014).

In this context, most studies in cardiomyopathies evaluate the effects of a treatment or therapy mainly on the left ventricle. Supporting our findings on the heart and skeletal muscle, a previous study demonstrated that 5 weeks of aerobic treadmill training also reduced cardiac fibrosis in animals with Chagas disease. However, that study utilized an experimental model of the chronic indeterminate form of the disease (Pedra-Rezende et al., 2021). In a study more similar to ours, the authors performed an 8-week treadmill aerobic training protocol in an experimental model of CCC, but in hamsters. They observed that the ExT protocol reduced cardiac fibrosis and inflammation, attenuated the progression of myocardial perfusion defects and increased the cardiorespiratory capacity of animals with CCC (Damasceno et al., 2024).

Another study also showed that 12-week treadmill ExT was able to reduce cardiac fibrosis, however, in an aged rat model. The authors explained that the mechanism responsible for reducing fibrosis in this study was the decrease in the expression of TGF-β and TIMP-1 with a reduction in the fibrosis content in the heart assessed by Masson’s trichrome staining after ExT (Kwak et al., 2011). The decrease in TGF-β expression was also identified in our study, however, we did not identify a statistical difference in the decrease in TIMP-1. Nevertheless, in the same line of this work, we also identified a reduction in the collagen content in the heart. In this way, ExT emerges as a promising treatment for reducing fibrosis in the heart and in other organs, and the probable molecular mechanisms are through the reduction of the expression of TGF-β, TIMP-1, TNF-α, Wnt/β-catenin and nuclear factor kappa-B (NF-kB), in addition to the reduction of reactive oxygen species (Ma et al., 2025).

The fact that ExT reduced cardiac fibrosis without altering the number of inflammatory cells also suggests a more refined mechanism of cardiac remodeling, in which collagen deposition and degradation are regulated independently of inflammatory infiltration. Although chronic inflammation in CCC is associated with extracellular matrix deposition and fibrosis, the presence of inflammatory cells does not necessarily imply fibrosis progression. ExT reduced the expression of the pro-fibrotic mediator TGF-β without affecting the total inflammatory burden in cardiac tissue. In other words, the absence of changes in the total number of inflammatory cells suggests that ExT may have induced an increase in the activity of these cells (Improta-Caria et al., 2021). To reinforce this hypothesis, one could discuss the possibility that ExT modulated macrophage polarization, increasing the M2/M1 ratio, which would favor a more anti-fibrotic environment without the need to alter the absolute number of inflammatory cells.

This specific modulation of extracellular matrix remodeling may occur through mechanisms independent of inflammatory infiltration, possibly involving the reduced expression of pro-fibrotic mediators such as TGF-β, as previously mentioned. The fact that the total number of inflammatory cells remained unchanged while cytokine levels (TNF-α, IL-6, and IL-1β) associated with chronic macrophage activation and myocardial damage in CCC were reduced, which suggests that ExT exerted a significant anti-inflammatory effect, likely due to a shift in the profile of these cells, promoting myocardial recovery. In summary, the results presented here strongly indicate that ExT in CCC has a positive functional and structural impact on the heart and reinforces the critical role of fibrosis in diastolic dysfunction in CCC. The improvement in diastolic function, as indicated by the decrease in IVRT after ExT, may be associated with reduced ventricular stiffness, which is in turn, consistent with lower myocardial fibrosis.

Evidence shows that cardiac fibrosis is an important factor in the development of arrhythmias (Kazbanov et al., 2016). Both *T. cruzi* and the inflammatory infiltrate in the heart favor the injury and death of cardiomyocytes, inducing increased collagen expression by cardiac fibroblasts at this site. Fibrosis can disrupt the propagation of electrical impulses in injured areas, creating a substrate that favors the development of cardiac arrhythmias, as observed in animals with CCC. So, the improvement in cardiac electrical disturbances after ExT protocol could result from appropriate ionic flow through cardiomyocytes due to fibrosis reduction. This result also corroborates the literature, as a study showed that aerobic physical training on a treadmill for 6 weeks promoted attenuation of ventricular arrhythmias in an experimental model of post-myocardial infarction mice (Qin et al., 2019).

The reverse of arrhythmias is crucial to Chagas disease patients since CCC is essentially an arrhythmogenic cardiopathy in which sudden death accounts for more than 50% of mortality. It can be observed that eight animals in the ExT CCC group developed monomorphic sustained ventricular tachycardia that was reverted in four animals after the ExT. Only one mouse with monomorphic sustained ventricular tachycardia evolved to total atrioventricular block. This is a very relevant result since monomorphic sustained ventricular tachycardia is associated with a very poor prognosis (Rassi et al., 2001). Furthermore, the main causes of sudden death are ventricular tachycardia (sustained or not) and fibrillation (Rassi et al., 2001).

In this context, regarding the importance of ExT, the gastrocnemius muscle was selected for analysis due to its large size and anatomical location in the posterior region of the mouse’s hind limbs. Together with the soleus muscle, it forms a muscle group that provides the propulsion force for walking, running and jumping. By contracting and relaxing these calf muscles, blood is pumped back to the heart with greater efficiency, improving venous return. The deterioration of gastrocnemius muscle in Chagas disease reduces the ability to perform physical exercise and compromises venous return, also contributing to worsening cardiac dysfunction. The reduction in skeletal muscle fibrosis area after ExT reinforces the beneficial effect of exercise in tissue remodeling.

Although our results strongly suggested that cardiac fibrosis reduction is the key factor for the reversal of monomorphic sustained ventricular tachycardia in these animals with CCC that performed ExT, the molecular mechanisms still need to be better elucidated in future studies. In this context, we propose that an immunomodulatory mechanism could be activated by the ExT based on gene expression results, such as reduced expression of TGF-β, TNF-α, IL-6, IL-1β and MMP-9 in the hearts of animals in the ExT CCC group compared to the Sed CCC group. This effect possibly favors the attenuation of cardiac remodeling, improving the conduction of the electrical impulse in the heart.

An increase in the number of inflammatory cells in the heart and skeletal muscle was observed in the Sed CCC and ExT CCC compared to the CTL group. However, no statistical differences were observed when comparing Sed CCC and ExT CCC. The duration of the ExT protocol probably was not sufficient to obtain a decrease in inflammation in the ExT CCC group.

The cytokines mentioned above are associated with inflammation and fibrosis and these two processes are closely linked to cardiac remodeling that leads to severe arrhythmias and mechanical dysfunction in CCC (Novaes et al., 2013; Nunes et al., 2018). Particularly, the increase in TGF-β expression is a critical factor in driving fibrosis in the heart. This fibrosis is linked to structural disorganization of myocytes, which in turn contributes to diastolic and/or systolic dysfunction, as well as electrical uncoupling between the ventricular muscle and the conduction system (Cevey et al., 2017; Nardi Gemme et al., 2022). TGF-β also induces many different effector processes related to different pathological states, such as atrial fibrillation (Dobaczewski et al., 2011), HF (Bocchi et al., 2017), promoting cardiac fibrosis in obesity (Improta-Caria et al., 2023), diabetes mellitus (Reeves and Andreoli, 2000), hypertension (Improta-Caria et al., 2021) and coronary arterial disease (Low et al., 2019). In CCC, TGF-β has increased expression and has been correlated to a poor prognosis (Araújo-Jorge et al., 2002; Ferreira et al., 2019), such as cardiac dysfunction, arrhythmias, ventricular dilation, increased risk of HF, and sudden death (Rassi et al., 2010).

Still regarding cardiac remodeling, MMPs are potential biomarkers for the progression of cardiomyopathy in chagasic patients. The role of MMP-9 in the development of CCC was demonstrated in a clinical trial, that showed a significant increase in MMP-9 in chagasic patients with EKG abnormalities and dilated cardiomyopathy (Bautista-López et al., 2013). Thus, the attenuation of MMP-9 gene expression after ExT may have contributed to the improvement of arrhythmias in mice with CCC, as observed in the present study.

Electrical disturbances in the heart can be attributed to many mechanisms besides fibrosis, such as alterations in ion channel gene expression or function. Ventricular myocytes enzymatically isolated from the heart of C57BL/6 mice infected by Colombian *T. cruzi* strain exhibited reduction in transient outward potassium current (Ito) (Roman-Campos et al., 2013). After a long period exposure to TNF-α, the authors reported an increase in nitric oxide and superoxide anion production, which in turn reduced Ito through reduced gene expression of Kv4.3 that encodes the potassium channel carrier (Fernández-Velasco et al., 2007; Gómez et al., 2008). Since Ito current contributes to early repolarization, the reduction in Ito augments the plateau phase of the action potentials, favoring early post depolarization occurrence, which in turn elevates the susceptibility to develop arrhythmias. In this context, we also identified an increased expression of TNF-α in the Sed CCC group, which potentially favored the development of arrhythmias. On the other hand, in mice from the ExT CCC group, a reduction in TNF-α expression was identified when compared to Sed CCC, which possibly favored the reversal of arrhythmias.

Another proinflammatory cytokine associated with arrhythmias is IL-1β, and its expression is also increased in chagasic mice as well as in patients with CCC (Petersen and Burleigh, 2003). Here, we demonstrated a reduction in gene expression of IL-1β in the ExT CCC compared to Sed CCC. During inflammatory processes, the effects of IL-1β include delay in action potential repolarization due to Ito reduction, that increases Ca^+2^ sparks, and the incidence of cardiac arrhythmias (Monnerat et al., 2016). The decrease in IL-1β levels after ExT, represents an additional potential effect to ameliorate cardiac arrhythmias.

In addition to IL-1β, we observed increased cardiac expression of IL-6 in Sed CCC mice when compared to CTL mice. This result is in accordance with the reported relevance of IL-6 in the progression of Chagas disease. Interestingly, the ExT CCC group had reduced IL-6 expression compared to Sed CCC and presented IL-6 levels closer to CTL group. The role of IL-6 in cardiac inflammation and fibrosis has already been proven in ischemic and infectious heart disease (Ponce et al., 2012; Fontes et al., 2015). In acute phase and in the CCC, IL-6 has long been recognized as a mediator of inflammatory cardiomyopathy, as well as, a promoter of endothelial dysfunction and myocardial fibrosis, which culminate in HF (Sunnemark et al., 1996; Diaz et al., 2009). Thus, another beneficial effect of ExT on CCC was to bring IL-6 levels closer to control group, which probably favored the improvement of arrhythmias as observed in this study.

ECHO analysis showed a decrease in isovolumetric relaxation interval after ExT which results in improved diastolic function. In line with these results, the fibrosis attenuation could be one of the determinant factors to cardiac function amelioration, which reduction could be linked to cardiac MMP-9 expression decrease in the ExT CCC in our study. Still regard to ECHO analysis, the comparative analyses between control and chagasic mice before training revealed significant differences in some parameters such as ventricular internal diameter during systole; posterior wall thickness during systole, ejection fraction; fractional shortening; end-diastolic volume and end-systolic volume. Together, these results could be misinterpreted, as the measured values were higher in the Sed CCC group compared to the CTL group. However, these data suggest a pseudo-normalization of cardiac function, rather than a true restoration. In diastolic dysfunction, the ventricular filling pattern may be altered, resulting in changes in the inflow of blood into the left ventricle detected by doppler. This can be observed in the initial stage of diastolic dysfunction by a prolonged relaxation phase of the left ventricle, a reduced early filling flow, and an increased late filling flow. On the other hand, in the pseudo normalized stage, an increase in the diastolic pressure of the left ventricle can be observed, which compensates for the abnormal relaxation and generates an apparent normality in the relation between the reduced early ventricular filling flow and the late ventricular filling flow. However, this scenario reflects a worsening of diastolic dysfunction.

An increase in ejection fraction and fractional shortening in chronically chagasic mice should also be interpreted with caution, as it may not reflect an actual improvement in cardiac function but rather a pathological adaptation to disease progression. This finding can be explained by pseudo-normalization, which masks the underlying cardiac dysfunction. The rationale for this finding may be associated with the progressive increase in ventricular stiffness and diastolic dysfunction. In more advanced stages, these two alterations can lead to compensatory hypertrophy and a reduction in end-diastolic volume, promoting an apparent elevation of systolic indices. Ventricular stiffness associated with fibrosis reduces ventricular compliance, impairing ventricular filling during diastole. Since ejection fraction and fractional shortening calculations are based on changes in diastolic and systolic volumes, a reduction in end-diastolic volume can lead to artificially increased percentage values of these indices, without a true improvement in contractility.

## 5 Conclusion

For the first time, it has been demonstrated that ExT promoted a reduction in the fibrosis area in both the heart and skeletal muscle of mice with CCC. This reduction contributed to an improvement in cardiac electrical conductance, mitigating the severity of arrhythmias. Additionally, ExT induced a decrease in the expression of proinflammatory and fibrotic genes in the heart. Thus, this study strongly suggests that ExT is a valuable non-pharmacological intervention for improving the progression of CCC.

## Data Availability

The raw data supporting the conclusions of this article will be made available by the authors, without undue reservation.
